# Intermittent Fasting Potentiates Aerobic Exercise to Reduce Hippocampal Amyloid Burden and Oxidative Stress via Suppression of NF‐κB/NLRP3 Signaling in an Aβ‐Injected Rat Model

**DOI:** 10.1155/omcl/9921337

**Published:** 2026-05-18

**Authors:** Taner Atasoy, Heidar Sajedi, Davar Khodadadi, Burak Tozoğlu, Mehmet Şirin Güler, Serkan Tevabil AKA, Mohammad Babaei

**Affiliations:** ^1^ Department of Exercise and Sports Sciences for Individuals With Disabilities, Istanbul Gelisim University, Istanbul, Türkiye, gelisim.edu.tr; ^2^ Department of Exercise and Sports Science for Disabled People, International Science and Technology University, Warsaw, Poland; ^3^ Sport Science Faculty, Department of Movement and Training Sciences, Duzce University, Duzce, Türkiye, duzce.edu.tr; ^4^ Department of Exercise Physiology, Islamic Azad University Central Tehran Branch, Tehran, Iran, iauctb.ac.ir; ^5^ Erzurum Provincial Directorate of Youth Services and Sports, Republic of Turkey Ministry of Youth and Sports, Erzurum, Türkiye; ^6^ Department of Physical Education and Sports, Agri Ibrahim Cecen University, Agri, Türkiye; ^7^ Department of Physical Education and Sport Teaching, Kafkas University, Sarıkamıs Campus, Kars, Türkiye, kafkas.edu.tr; ^8^ Department of Physical Education and Sports Sciences, University of Mohaghegh Ardabili, Ardabil, Iran, uma.ac.ir

**Keywords:** aerobic exercise, Alzheimer’s disease, cognitive function, hippocampus, intermittent fasting, NLRP3 inflammasome, β-hydroxybutyrate

## Abstract

NOD‐like receptor protein 3 (NLRP3) inflammasome‐driven neuroinflammation contributes to Alzheimer’s disease (AD) progression, yet effective strategies to target this pathway are limited. We investigated whether aerobic exercise performed in a fasted state, rather than the fed state, would potentiate β‐hydroxybutyrate (BHB)‐dependent inhibition of NLRP3 inflammasome signaling. Twenty‐month‐old male Wistar rats were randomly assigned to five groups: AD, AD + intermittent fasting (ADIF), AD + aerobic exercise (ADAE), ADIF + aerobic exercise (ADIFAE), and sham‐injected control (SC). AD‐like pathology was induced by bilateral intrahippocampal injection of amyloid‐β (Aβ)_1–42_. The IF regimen consisted of a daily 14‐h fast (06:00–20:00). Exercise consisted of moderate‐intensity treadmill running (5 days/week for 4 weeks), either in the fed state or after ∼12.5 h of fasting. Aβ injection impaired spatial learning and memory, elevated soluble Aβ_1–42_ (sAβ), malondialdehyde (MDA), NF‐κB, NLRP3, caspase‐1, interleukin‐1β (IL‐1β), and IL‐18, and reduced superoxide dismutase (SOD) activity and brain‐derived neurotrophic factor (BDNF) expression in the hippocampus (*p*  < 0.05). Both IF and exercise partially reversed cognitive impairments by reducing sAβ and oxidative stress, increasing BHB, suppressing NF‐κB/NLRP3 signaling, and restoring BDNF (*p*  < 0.05), while fasted‐state exercise produced significantly larger effects than either intervention alone (*p*  < 0.05). Our findings suggest that performing exercise in a fasted state provides complementary metabolic, anti‐inflammatory, and cognitive benefits that exceed those of either intervention alone. This combined regimen may represent a promising nonpharmacological strategy for targeting metabolic‐immune and neurotrophic pathways relevant to AD progression.

## 1. Introduction

Alzheimer’s disease (AD) is a progressive neurodegenerative disorder characterized by extracellular amyloid‐β (Aβ) plaques, intracellular neurofibrillary tangles of hyperphosphorylated tau, and widespread neuronal loss [1]. Beyond these hallmark lesions, chronic neuroinflammation has emerged as a central driver of AD pathogenesis. In particular, the NOD‐like receptor protein 3 (NLRP3) inflammasome, an innate immune sensor expressed in microglia and astrocytes, has been implicated as a key mediator of AD‐related inflammation [2]. Activation of NLRP3 by aggregated Aβ and pathological tau triggers caspase‐1‐dependent maturation and release of interleukin‐1β (IL‐1β) and IL‐18, which suppress brain‐derived neurotrophic factor (BDNF) signaling, promote synaptic dysfunction, and accelerate neuronal loss and cognitive decline [3, 4]. Elevated NLRP3 signaling in AD brain tissue is therefore believed to perpetuate a chronic inflammatory state that contributes to disease progression [5]. Consequently, NLRP3 represents an attractive therapeutic target, and interventions that attenuate its activity hold promise for slowing neurodegeneration [2, 5].

Given the limited efficacy and adverse effects of many pharmacologic approaches, lifestyle interventions that broadly modulate metabolic, inflammatory, and neurotrophic pathways have gained attention. Regular aerobic exercise is well‐recognized for its neuroprotective and anti‐inflammatory effects in aging and AD. In animal models, moderate treadmill training improves spatial learning and memory, reduces Aβ burden, lowers oxidative stress, and shifts neuroinflammatory phenotypes toward a less damaging profile [6, 7]. Notably, recent work reports that long‐term treadmill running reduces hippocampal NLRP3 and IL‐1β expression in transgenic AD mice, linking physical activity to direct suppression of inflammasome‐mediated signaling [8]. These effects are mediated, at least in part, by enhanced neurotrophic support (e.g., increased BDNF), improved mitochondrial function, and activation of endogenous antioxidant defenses.

Intermittent fasting (IF) and other ketogenic approaches are complementary lifestyle strategies that also exert neuroprotective effects. IF includes dietary regimens that cycle between periods of fasting and feeding (e.g., alternate‐day fasting or time‐restricted feeding). A growing body of preclinical evidence suggests that IF can protect against neurodegenerative processes [9, 10]. Several animal studies have demonstrated that IF reduces the accumulation of Aβ plaques and slows cognitive decline [11–13]. IF induces a metabolic switch to increased fat oxidation and ketogenesis, resulting in a decrease in glucose and insulin levels while circulating free fatty acids and ketone bodies, especially β‐hydroxybutyrate (BHB), increase [14].

This fasting‐induced ketosis has direct anti‐inflammatory effects [15]. It was shown that acute fasting or ketone treatments reduce components of the NLRP3 pathway, such as NLRP3 expression, caspase‐1 activity, and IL‐1β/IL‐18 release [16]. Shippy et al. [17] found that brains from AD patients contain lower BHB and that BHB supplementation in an AD mouse model inhibited NLRP3 signaling and reduced Aβ deposition. Another study by Traba et al. [18] demonstrated that 24‐h fasting renders monocyte NLRP3 less responsive to activating stimuli via SIRT3‐dependent mechanisms. These reports indicate that fasting‐related metabolic changes can enhance neurotrophic signaling via BDNF upregulation while simultaneously suppressing maladaptive innate immune activation in the brain [19, 20].

Although aerobic exercise and IF each mitigate AD‐related cognitive deficits and neuroinflammation through partly overlapping mechanisms [8, 20], their combined effects remain poorly characterized. From a mechanistic standpoint, exercising in a fasted state could enhance the metabolic switch and increase BHB to levels greater than those achieved by fed‐state exercise, thus potentiating BHB‐mediated inhibition of NLRP3 while simultaneously engaging exercise‐dependent neurotrophic and antioxidant pathways. Translationally, this question is important because exercise timing and meal patterns are modifiable behaviors that could be optimized to maximize neuroprotective benefit.

Accordingly, we hypothesized that moderate‐intensity aerobic exercise performed after prolonged fasting would elevate hippocampal BHB to a greater extent than normal fed‐state exercise, leading to stronger suppression of NLRP3 inflammasome signaling, reduced oxidative stress, higher BDNF expression, and improved hippocampal‐dependent spatial memory in a rat model of Aβ‐induced neurotoxicity.

## 2. Methods

### 2.1. Animals

Sixty 20‐month‐old male Wistar rats were obtained from the Animals Center of Pasteur Institute (Tehran, Iran) and were assigned to five groups (*n* = 12 each group): AD, AD + IF (ADIF), AD + aerobic exercise (ADAE), ADIF + aerobic exercise training (ADIFAE), and sham‐injected control (SC). Before the group assignment, all rats underwent a 1‐week familiarization period. It consisted of three treadmill running sessions (10 min/session, 5 m/min, 0% incline) in a fasted state (∼12.5 h before each session). Animals were housed in groups (three rats per cage) under controlled conditions (22 ± 1°C, 50%–60% humidity, 12/12 h light/dark cycle). All animal care and experimental procedures were conducted in accordance with the National Institutes of Health guidelines and were approved by the Research Ethics Committee of Islamic Azad University, Central Tehran Branch (IR.IAU.CTB.REC.1400.015).

### 2.2. Surgery

Human Aβ_1–42_ (Abcam, Germany) was prepared following Khodadadi et al. [6]. Aβ was dissolved in 3% DMSO (5 μg/μL), aliquoted (30 μL/vial), and stored at −80°C. To induce fibrillization, aliquots were incubated at 37°C for 7 days. Animals were anesthetized with ketamine/xylazine (100/25 mg/kg, i.p.) and fixed in a stereotaxic frame (Stoelting, USA). After a midline scalp incision, burr holes were drilled bilaterally. Using Paxinos and Watson [21] coordinates (AP: −3.8 mm, ML: ±2.2 mm, DV: −2.7 mm from bregma), 2 μL of fibrillized Aβ solution (5 μg/μL) was injected into each hippocampal CA1 subregion via a Hamilton syringe (right and left hemispheres; 10 μg total Aβ/rat). SC animals were injected with DMSO using the same procedure. Postsurgery, animals recovered in individual cages for 1 week with monitoring.

### 2.3. Animal Feeding and IF Administration

All animals had access to standard rodent chow (18.2 kcal% fat, energy 3.5 kcal/g, P1103F‐25, SLACOM, Shanghai, China) and water ad libitum until the initiation of IF and/or exercise interventions. 1‐week postsurgery, rats assigned to the ADIF and ADIFAE groups began a 4‐week IF protocol. Daily IF consisted of a 14‐h fast (06:00–20:00) followed by a 10‐h feeding during the active dark phase (ZT12–ZT22; ZT0 = lights on). Water remained available ad libitum. This regimen was selected for its established neuroprotective effects [22, 23] and ability to elevate BHB in rodents [23–25]. The SC, AD, and ADAE groups had diet and water access ad libitum throughout the study.

### 2.4. Aerobic Exercise Protocol

Seven days postsurgery, rats in exercise groups started a 4‐week treadmill running program (5 days/week). Training followed a progressive protocol: weeks 1–2, two 15‐min runs at 10 m/min, separated by 3‐min walks at 5 m/min; week 3, three 15‐min runs at 15 m/min, separated by 3‐min walks at 5 m/min; week 4, four 15‐min runs at 15 m/min, separated by 3‐min walks at 5 m/min [26]. Sessions began at 18:30. The ADIF group underwent a 12.5‐h fast prior to the session, while the ADAE group had ad libitum access to food and water. Each session included a 3‐min warm‐up and cool‐down, both performed at a speed of 5 m/min and 0% incline. Nonexercise animals (AD, ADIF, and SC) were placed daily on an inactive treadmill under identical conditions without enforced running to control for handling/environmental stress.

### 2.5. The Morris Water Maze Test

Spatial learning and memory were assessed using the Morris Water Maze [27]. Rats underwent habituation (60‐s free swim without a platform) 24 h before testing. The maze consisted of a black circular pool (136 cm diameter and 60 cm depth) filled with water (23–25°C, maintained throughout) within a cue‐rich room. Four virtual quadrants (NE, NW, SE, and SW) were defined. A transparent platform (submerged 1 cm) was fixed in the NW (target) quadrant.

Acquisition phase (4 days). Each day comprised one block of four trials (max 90s/trial). Rats started randomly at one quadrant facing the wall. Upon finding the platform, they remained for 20 s before the next trial; if unsuccessful after 90s, they were guided to it and rested for 20 s. Escape latency was recorded (EthoVision XT7, Noldus, Netherlands). The interval between the blocks was ∼24 h for each animal. All trials were conducted at roughly the same time each day (between 8:00 a.m. and 12:00 p.m.) in order to minimize variability in performance due to time of day.

Probe test (24 h postacquisition). The platform was removed. Rats started in the quadrant opposite the target (SE) and swam freely for 60 s. The percent of time spent in the target quadrant was recorded (memory retention).

Visible‐platform test (1‐h postprobe). To control for visual–motor deficits, one block of four trials was conducted with a foil‐covered platform elevated ∼1.5 cm above water in the SE quadrant, and escape latency was recorded.

### 2.6. Sampling

For hippocampal soluble Aβ_1–42_ (sAβ) and BHB quantification, five randomly selected rats per group were anesthetized (ketamine/xylazine: 100/10 mg/kg, i.p.) 60 min after the last training session and sacrificed. The remaining animals (*n* = 7 per group) were also sacrificed 24 h postbehavioral testing to assay neuroinflammation, oxidative stress and neurotrophic factors. The 60‐min postexercise BHB measurement directly tests our hypothesis regarding the role of acute BHB elevation, while the later 24 h postbehavioral testing measurement provides insight into the potential cellular adaptations following this elevation. Brains were rapidly extracted; hippocampi were dissected on ice, flash‐frozen in liquid N_2_, and stored at −80°C pending analysis.

### 2.7. Western Blotting Assay

Hippocampal tissues were homogenized in ice‐cold RIPA buffer (Cell Signaling Technology) supplemented with protease and phosphatase inhibitor cocktails. Lysates were centrifuged at 12,000 × *g* for 15 min at 4°C, and the supernatant was collected for protein quantification using the bicinchoninic acid (BCA) assay. Equal amounts of protein (20–30 µg per lane) were mixed with Laemmli sample buffer, denatured at 95°C for 5 min, resolved on 10%–12% SDS–PAGE gels, and transferred to PVDF membranes using a semi‐dry transfer system. Membranes were blocked for 1 h at room temperature in 5% nonfat dry milk in tris‐buffered saline with 0.1% Tween‐20 (TBST), then incubated overnight at 4°C with primary antibodies diluted in blocking buffer: NF‐κB p65 (1:1000; ab16502, Abcam), NLRP3 (1:1000; ab263899, Abcam), caspase‐1 (1:1000; MA5‐16215, Thermo Fisher Scientific), IL‐1β (1:1000; ab254360, Abcam), IL‐18 (1:1000; ab191860, Abcam), and BDNF (1:500; ab108319, Abcam). After three 10‐min washes in TBST, membranes were incubated with the appropriate HRP‐conjugated secondary antibody (1:5000; Abcam) for 2 h at room temperature. Following additional washes, immunoreactive bands were visualized by enhanced chemiluminescence and imaged on a digital detection system. Band intensities were quantified by densitometry using ImageJ and normalized to β‐actin (1:1000; ab227387, Abcam). All experiments were performed in duplicate.

### 2.8. Measurement of Soluble Aβ_1–42_ Concentration and BHB

Hippocampal sAβ (CSB‐E10786r, Cusabio Biotech) and BHB (ab83390, Abcam) were quantified using the commercial ELISA kit following the manufacturer’s instructions. Briefly, hippocampi were homogenized in 1.0 mL phosphate‐buffered saline (PBS), subjected to two freeze–thaw cycles, and centrifuged at 5000 × *g* for 5 min at 4°C. Supernatants were collected, and total protein concentration was determined. Equal amounts of protein were loaded into antibody‐coated microplate wells together with standards. Plates were incubated at 37°C for 1 h, washed, incubated with the HRP‐conjugated detection antibody for 1 h, washed again, and developed with TMB substrate in the dark. Reactions were stopped with the provided acid stop solution, and absorbance was measured at 450 nm. Concentrations of sAβ and BHB were interpolated from the standard curves and normalized to total protein. Samples were assayed in duplicate.

### 2.9. Measurement of Oxidative Stress Levels

Malondialdehyde (MDA) was measured using the thiobarbituric acid (TBA) reactive substances (TBARS) assay kit (ab118970, Abcam). Hippocampal tissue was homogenized in the kit‐supplied butanol‐containing buffer, centrifuged, and the supernatant was collected. Samples were reacted with the TBA reagent under acidic conditions and heated at 95°C to form MDA–TBA adducts. Following cooling and clarification as instructed by the manufacturer, absorbance was measured at 532 nm, and MDA concentrations were interpolated from the supplied standard curve. Superoxide dismutase (SOD) activity was determined using the nitroblue tetrazolium (NBT) reduction assay kit (ab65354, Abcam). Hippocampal lysates were incubated with the kit reaction mix containing xanthine oxidase and NBT; SOD inhibits the reduction of NBT to formazan. The change in NBT formazan absorbance was read at 560 nm, and SOD activity was calculated from the kit calibration curve and expressed as units per mg total protein. All assays were performed in duplicate, and values were normalized to total protein.

### 2.10. Statistical Analysis

Data distribution was assessed with the Shapiro–Wilk test and homogeneity of variances with Levene’s test. Escape latency during the Morris Water Maze acquisition was averaged by day for each animal and analyzed with a 4 × 5 mixed model repeated‐measures ANOVA (day as the within‐subjects factor and group/treatment as the between‐subjects factor). A two‐way ANOVA was used to analyze body weight, probe, visible‐platform, and biochemical measures across groups. When ANOVA indicated a significant main effect, pairwise comparisons were performed using Fisher’s least significant difference (LSD) post hoc test. Analyses were conducted in IBM SPSS Statistics v27, and significance was set at *p*  < 0.05. Results are presented as mean ± SD.

## 3. Results

Consistent with prior reports [6, 26], our pilot study revealed that bilateral intrahippocampal injection of Aβ_1–42_ led to impairments in spatial learning and memory. 1‐week after injection, Aβ_1–42_‐infused rats (*n* = 7) showed significantly increased escape latencies during acquisition and spent significantly less time in the target quadrant during the probe trial compared with age‐matched controls (*n* = 7; *p*  < 0.001). These deficits persisted for at least 6 weeks (data not shown).

**Figure 1:**
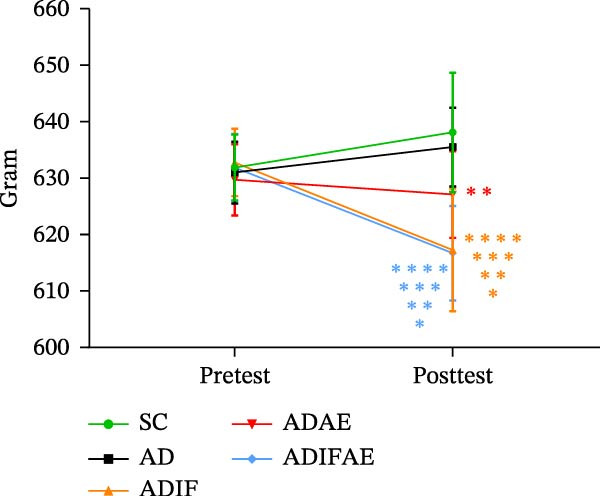
Intermittent fasting, exercise in the fed state, and exercise in the fasted state reduce body weight in the Aβ‐injected rat model of AD (*n* = 12 per group).  ^∗^ *p*  < 0.001 significant difference from pretest.  ^∗∗^ *p*  < 0.05 significant difference from SC.  ^∗∗∗^ *p*  < 0.001 significant difference from AD.  ^∗∗∗∗^ *p*  < 0.05 significant difference from ADAE.

Body weight analysis showed main effects of time (*F* = 18.354, *p*  < 0.001) and group (*F* = 5.178, *p*  < 0.01), with a significant interaction (*F* = 19.521, *p*  < 0.001). After the interventions, rats in the IF groups lost significantly more weight compared to their baselines, controls (*p*  < 0.001), and ADAE group (*p*  < 0.05). The body weight of ADAE animals was also lower compared to SC and AD groups (*p*  < 0.05; Figure 1).

**Figure 2:**
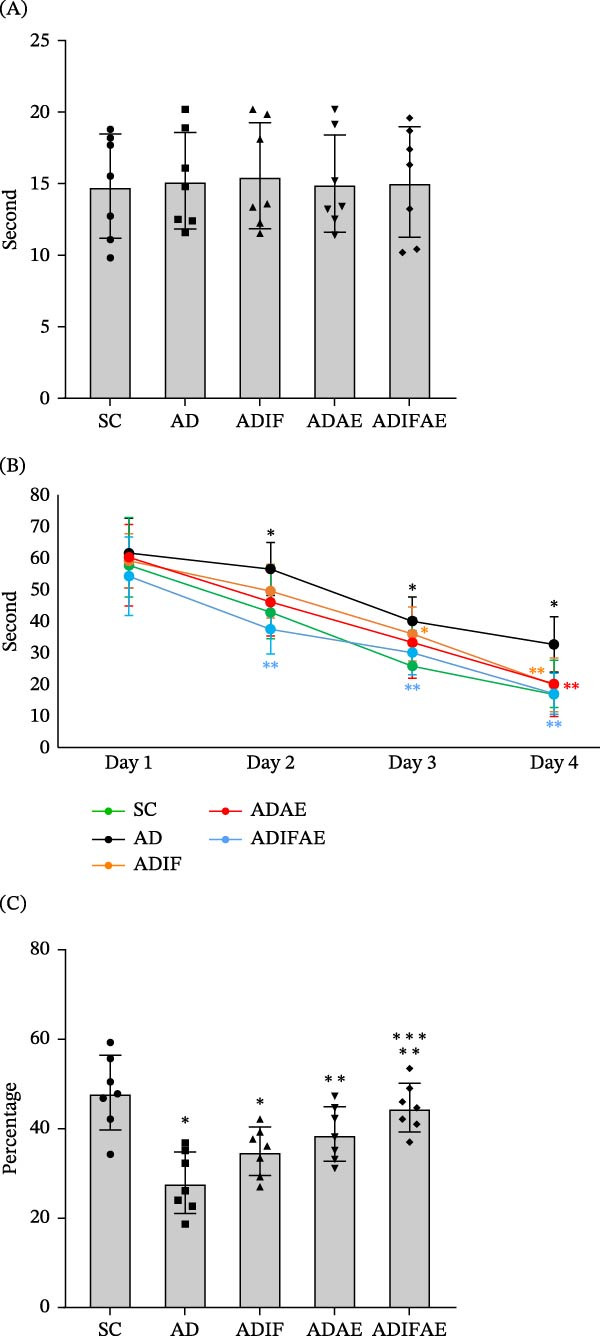
Intermittent fasting, exercise in the fed state, and exercise in the fasted state improve spatial learning and memory in the Aβ‐injected rat model of AD (*n* = 7 per group). (A) Time to find the platform in the visible test. (B) Escape latency in the acquisition phase. (C) The percent of time spent in the target quadrant in the probe trial.  ^∗^ *p*  < 0.05 significant difference from SC.  ^∗∗^ *p*  < 0.05 significant difference from AD.  ^∗∗∗^ *p*  < 0.05 significant difference from ADIF.

There were no significant effects of IF (*F* = 0.029, *p*  > 0.05), exercise (*F* = 0.056, *p*  > 0.05), or their interaction (*F* = 0.007, *p*  > 0.05) on the visible‐platform test, indicating comparable sensorimotor and visual ability across groups (Figure 2A). A mixed model repeated‐measures ANOVA on acquisition escape latency revealed significant main effects of time (*F* = 137.914, *p*  < 0.001) and group (*F* = 6.033, *p*  < 0.001), whereas the time × group interaction was not significant (*F* = 0.882, *p* = 0.524). There were no between‐group differences on day 1 (*p*  > 0.05). Post hoc comparisons showed that the AD group exhibited significantly longer escape latencies than the SC group on days 2–4 (*p*  < 0.05). The ADIF group also showed a significantly prolonged escape latency relative to SC animals on day 3 (*p*  < 0.05). Compared with the AD group, the ADIFAE group displayed significantly shorter escape latencies on days 2–4 (*p*  < 0.05). In addition, both the ADIF and ADAE groups demonstrated significantly reduced escape latency relative to the AD group on day 4 (*p*  < 0.05; Figure 2B).

Two‐way ANOVA revealed significant main effects of IF (*F* = 8.190, *p*  < 0.01) and exercise (*F* = 20.755, *p*  < 0.001) on spatial memory performance, but there was no significant IF × exercise interaction (*F* = 0.064, *p*  > 0.05). Post hoc comparisons indicated that the percentage of time spent in the target quadrant was significantly less in the AD (*p*  < 0.001) and ADIF (*p*  < 0.05) groups than in the SC rats. Exercise alone (*p*  < 0.05) and combined IF plus exercise (*p*  < 0.001) produced greater percentages of time in the target quadrant than the AD group. Additionally, the ADIFAE group spent a significantly higher percentage of time in the target quadrant than the ADIF group (*p*  < 0.05; Figure 2C).

**Figure 3:**
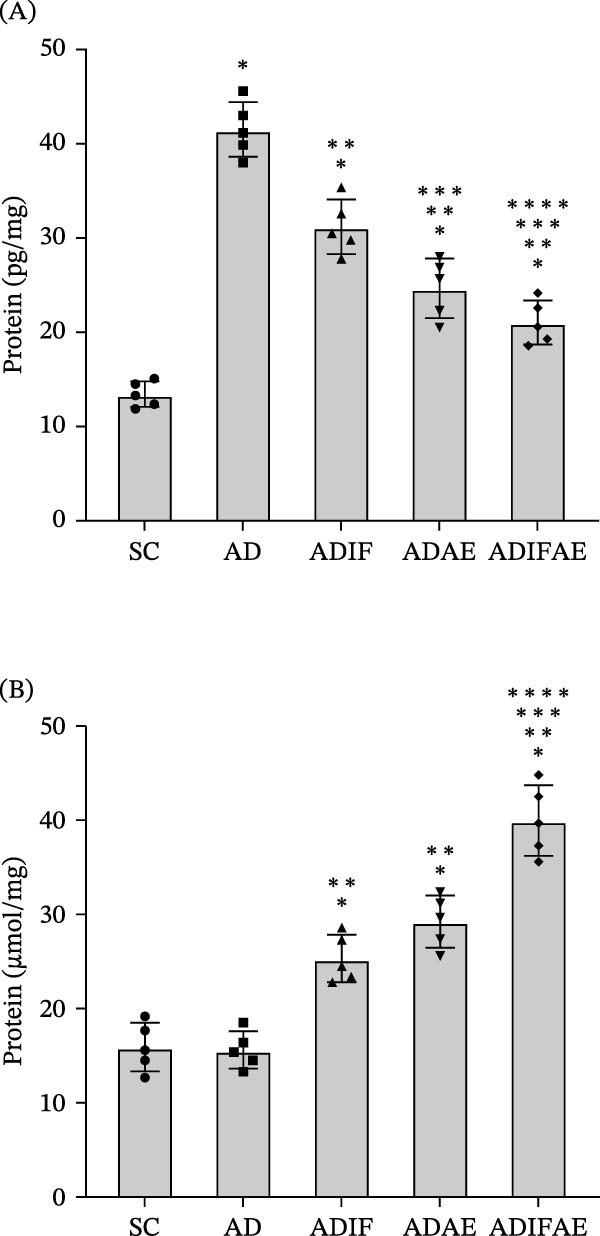
Intermittent fasting, exercise in the fed state, and exercise in the fasted state modulate hippocampal soluble Aβ_1–42_ (A) and β‐hydroxybutyrate (B) concentration in the Aβ‐injected rat model of AD (*n* = 5 per group).  ^∗^ *p*  < 0.05 significant difference from SC.  ^∗∗^ *p*  < 0.05 significant difference from AD.  ^∗∗∗^ *p*  < 0.05 significant difference from ADIF.  ^∗∗∗∗^ *p*  < 0.05 significant difference from ADAE.

Two‐way ANOVA revealed a significant effect of IF (*F* = 30.062, *p*  < 0.01), exercise (*F* = 112.946, *p*  < 0.001) and IF × exercise interaction (*F* = 6.945, *p*  < 0.05) on hippocampal sAβ. Post hoc comparisons showed that all Aβ‐injected groups had higher sAβ than SC animals (*p*  < 0.001). Relative to the untreated AD group, sAβ was significantly reduced in the ADIF, ADAE, and ADIFAE groups (*p*  < 0.001). ADIFAE had lower sAβ than ADIF (*p*  < 0.001) and ADAE (*p*  < 0.05). Furthermore, the sAβ in ADAE was lower than ADIF (*p*  < 0.001; Figure 3A).

There were also significant effects for IF (*F* = 65.318, *p*  < 0.001) and exercise (*F* = 125.457, *p*  < 0.001) for hippocampal BHB (*F* = 66.921, *p*  < 0.001), but their interaction was not significant (*F* = 0.163, *p*  > 0.05). IF, aerobic exercise, and IF plus aerobic exercise increased hippocampal BHB relative to SC and AD (*p*  < 0.001). The ADIFAE group exhibited higher BHB than either ADIF or ADAE (*p*  < 0.001; Figure 3B).

**Figure 4:**
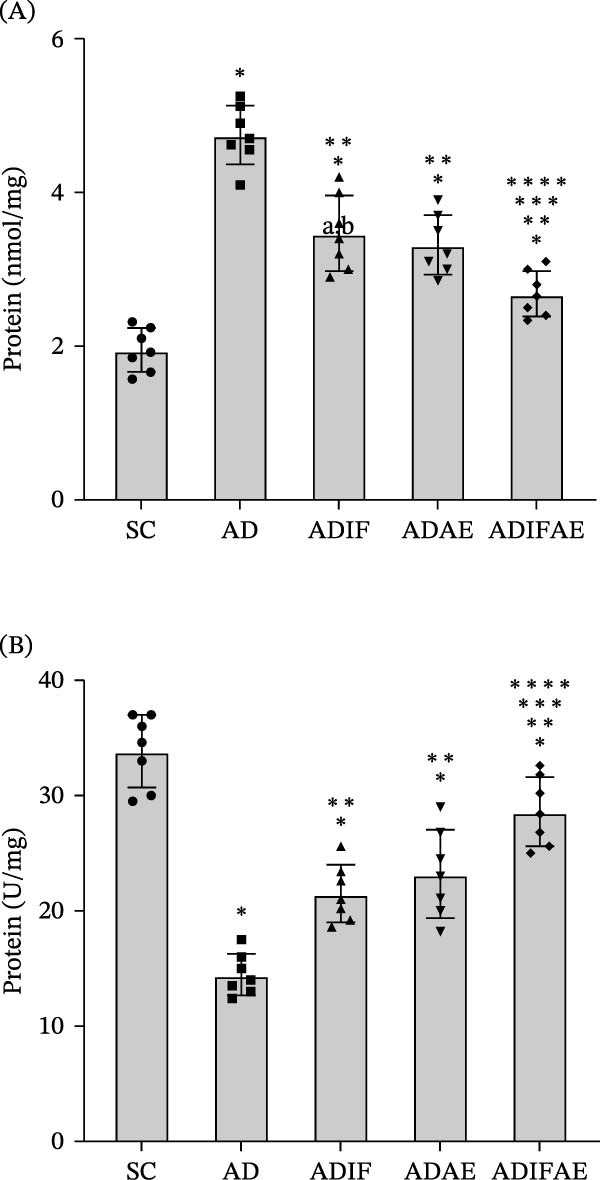
Intermittent fasting, exercise in the fed state, and exercise in the fasted state mitigate oxidative stress in the hippocampus of an Aβ‐injected rat model of AD (*n* = 7 per group). (A) MDA level. (B) SOD activity.  ^∗^ *p*  < 0.01 significant difference from SC.  ^∗∗^ *p*  < 0.001 significant difference from AD.  ^∗∗∗^ *p*  < 0.001 significant difference from ADIF.  ^∗∗∗∗^ *p*  < 0.01 significant difference from ADAE.

For oxidative stress and antioxidant capacity, two‐way ANOVAs showed significant main effects for MDA level (IF: *F* = 40.980, *p*  < 0.001, and exercise: *F* = 54.819, *p*  < 0.001) and SOD activity (IF: *F* = 32.653, *p*  < 0.001, and exercise: *F* = 53.154, *p*  < 0.001). The IF × exercise interaction reached significance only for MDA (*F* = 4.594, *p*  < 0.05), but not for SOD activity (*F* = 0.561, *p*  > 0.05). Hippocampal MDA was elevated in all Aβ‐injected groups compared with SC animals (*p*  < 0.001). The AD group displayed higher MDA than the ADIF, ADAE, and ADIFAE groups (*p* < 0.001). MDA in the ADIFAE group was lower than in the ADIF (*p*  < 0.001) and ADAE (*p*  < 0.01) groups, indicating the greatest reduction in lipid peroxidation with combined treatment. On the other hand, SOD activity was lower in the Aβ‐injected groups than in the SC animals (*p*  < 0.01). The AD group had lower SOD activity than ADIF, ADAE, and ADIFAE (*p*  < 0.001). The SOD activity in ADIF (*p*  < 0.001) and ADAE (*p*  < 0.01) groups was significantly lower than in ADIFAE (Figure 4).

**Figure 5:**
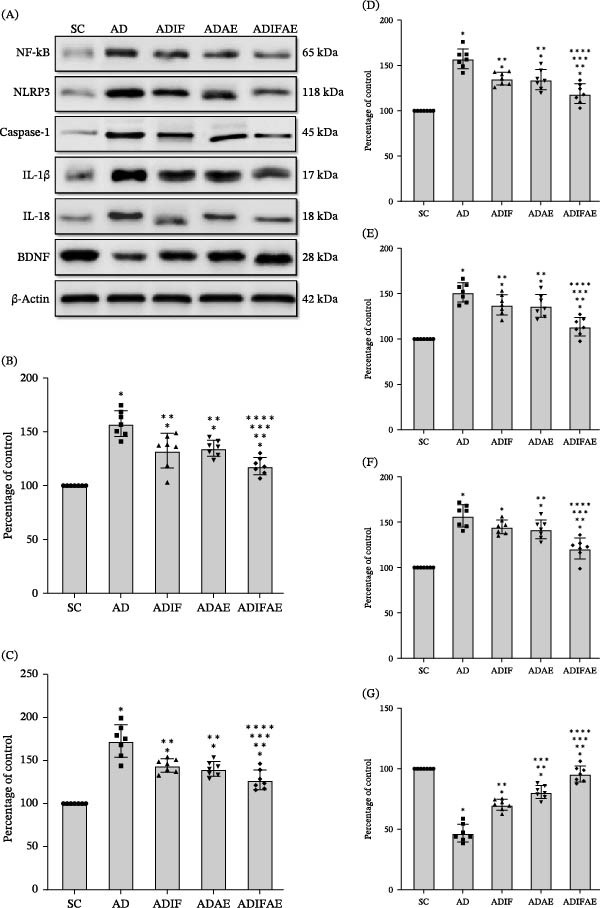
Intermittent fasting, exercise in the fed state, and exercise in the fasted state modify NLRP3 inflammasome signaling and BDNF expression in the hippocampus of an Aβ‐injected rat model of AD (*n* = 7 per group). (A) Representative western blots. (B) The relative protein expression of NF‐κB. (C) The relative protein expression of NLRP3. (D) The relative protein expression of caspase‐1. (E) The relative protein expression of IL‐1β. (F) The relative protein expression of IL‐18. (G) The relative protein expression of BDNF.  ^∗^ *p*  < 0.05 significant difference from SC.  ^∗∗^ *p*  < 0.001 significant difference from AD.  ^∗∗∗^ *p*  < 0.001 significant difference from ADIF.  ^∗∗∗∗^ *p*  < 0.05 significant difference from ADAE.

Two‐way ANOVAs revealed significant main effects of IF and exercise for hippocampal NF‐κB (IF: *F* = 23.193, *p*  < 0.001; exercise: *F* = 18.318, *p*  < 0.001), NLRP3 (IF: *F* = 18.796, *p*  < 0.001; exercise: *F* = 26.815, *p*  < 0.001), caspase‐1 (IF: *F* = 23.713, *p*  < 0.001; exercise: *F* = 26.813, *p*  < 0.001), IL‐1β (IF: *F* = 18.751, *p*  < 0.001; exercise: *F* = 20.999, *p*  < 0.001), and IL‐18 (IF: *F* = 16.626, *p*  < 0.001; exercise: *F* = 22.634, *p*  < 0.001). However, the IF × exercise interactions were not significant for any marker (NF‐κB: *F* = 0.967; NLRP3: *F* = 2.638; caspase‐1: *F* = 0.712; IL‐1β: *F* = 1.187; IL‐18: *F* = 1.023; *p*  > 0.05 for all). Aβ injection significantly upregulated components of the NLRP3 inflammasome pathway compared with the SC group (*p*  < 0.05). IF produced significant reductions in NF‐κB, NLRP3, caspase‐1, and IL‐1β versus the AD group, whereas the reduction in IL‐18 did not reach significance (*p* = 0.052). Aerobic exercise and the combined IF plus aerobic exercise regimen each significantly lowered all measured inflammasome markers relative to AD (*p*  < 0.05). Moreover, the ADIFAE group exhibited significantly greater suppression of NLRP3 inflammasome signaling than either ADIF or ADAE (*p*  < 0.05), indicating the largest anti‐inflammatory effect with the combined treatment (Figure 5, Figures S1 and S2).

Hippocampal BDNF showed significant main effects of IF (*F* = 70.523, *p*  < 0.001) and exercise (*F* = 169.016, *p*  < 0.001). The IF × exercise interaction did not reach statistical significance (*F* = 3.366, *p*  > 0.05). Aβ injection produced a significant reduction in BDNF in the AD, ADIF, and ADAE groups compared with SC (*p*  < 0.001). IF, aerobic exercise, and their combination each partially restored BDNF levels relative to AD (*p*  < 0.001). The combined intervention elicited larger increases in BDNF than either ADIF or ADAE alone (*p*  < 0.001), and aerobic exercise produced a greater BDNF increase than fasting alone (*p*  < 0.001; Figure 5G).

## 4. Discussion

The multifactorial etiology of AD has limited the success of single‐target therapies, prompting interest in multitarget strategies that address several pathogenic processes simultaneously [28]. Based on this rationale, we investigated whether performing moderate aerobic exercise in a fasted state, rather than the normal fed state, would potentiate BHB‐dependent inhibition of NLRP3 inflammasome signaling and thereby improve hippocampal synaptic plasticity and spatial cognition in an Aβ_1–42_ rat model. Intrahippocampal Aβ produced spatial learning and memory deficits accompanied by elevated sAβ, increased oxidative stress, activation of NF‐κB/NLRP3 signaling, and reduced hippocampal BDNF. Both IF and aerobic exercise partially reversed these impairments by reducing sAβ and oxidative stress, increasing hippocampal BHB, suppressing NF‐κB/NLRP3 signaling, and restoring BDNF and cognitive performance. Importantly, our behavioral and biochemical data indicated both additive and synergistic benefits when aerobic exercise is performed in a fasting state. Significant IF × exercise interactions for hippocampal sAβ and MDA indicate that the combined treatment produced synergistic reductions in amyloid burden and lipid peroxidation. By contrast, cognitive outcomes, inflammatory markers, and BDNF showed no statistically significant interaction despite larger mean improvements in the ADIFAE group, consistent with largely additive effects for those endpoints. To our knowledge, this is the first report showing that fasted‐state exercise enhances NLRP3‐dependent anti‐inflammatory and neurotrophic responses in an Aβ‐based rodent model.

While intrahippocampal Aβ injection does not capture the full complexity or chronicity of the human AD, particularly the tau component, our data confirm that acute injection successfully induces key hallmarks of AD, including spatial learning and memory impairments, elevated sAβ burden, increased oxidative stress, and activation of the neuroinflammatory cascade. There is substantial evidence supporting the view that soluble, oligomeric forms of Aβ, as their elevated levels revealed in the present study, are more critical than aggregated plaques in driving AD‐like cognitive deficits by directly harming synapses, and their levels correlate more closely with memory impairment than plaque burden [29]. The observed upregulation of hippocampal NF‐κB, NLRP3, caspase‐1, IL‐1β, and IL‐18 aligns with studies suggesting that the NLRP3 inflammasome is a critical stimulus of AD pathogenesis [30, 31]. The canonical pathway involves Aβ uptake by microglia, leading to reactive oxygen species (ROS)‐dependent activation of NF‐κB, which stimulates NLRP3 expression. Subsequent NLRP3 oligomerization catalyzes the cleavage of procaspase‐1 into active caspase‐1, which then processes pro‐IL‐1β and pro‐IL‐18 into their mature, highly inflammatory forms [32]. The reduction in BDNF we observed in the AD group is a critical downstream consequence of this inflammatory environment, as IL‐1β is known to negatively regulate BDNF expression, thereby impairing synaptic plasticity and neuronal survival [4].

The partial reversal of these deficits by either IF or aerobic exercise alone in the present study underscores the therapeutic potential of lifestyle interventions. The beneficial effects of exercise on brain health are multifaceted, including enhanced neurogenesis, synaptic plasticity, and antioxidant defense mechanisms [33]. Regular aerobic exercise has also been shown to reduce neuroinflammation in various AD models [34]. For example, Khodadadi et al. [6] demonstrated that treadmill running decreased hippocampal amyloid burden by enhancing the clearance of both soluble and aggregated Aβ in the Aβ_1–42_‐injected rat model of AD. Choi et al. [35] reported that aerobic exercise can enhance the cognitive functions of LPS‐treated mice by suppressing microglia‐mediated neuroinflammation through its impact on the expression of BDNF/FNDC5/irisin. In addition, Li et al. [8] found that long‐term exercise training reduces microglial activation, NLRP3‐mediated neuroinflammatory responses, and hippocampal neuronal damage in APP/PS1 mice. Consistent with these reports, our study revealed that short‐term aerobic treadmill running ameliorates cognitive decline by downregulating the sAβ, oxidative stress, and NF‐κB/NLRP3 signaling pathway in an Aβ model of AD.

Similarly, the benefits of IF on AD are well‐documented and are largely attributed to metabolic switching from glucose to ketone bodies as a primary energy source [19]. BHB is not only an alternative fuel but also a signaling molecule with potent anti‐inflammatory and neuroprotective properties [36]. It had been demonstrated that BHB administration reduced plaque formation, microgliosis, apoptosis‐associated speck‐like protein containing a caspase recruitment domain speck formation, and caspase‐1 activation in the 5xFAD mouse model of AD [17]. Another study found that BHB administration has a neuroprotective effect in BV2 microglial cells by inducing microglial polarization towards an M2 anti‐inflammatory phenotype, reducing migratory capacity and pro‐inflammatory cytokine IL‐17, and increasing anti‐inflammatory cytokine IL‐10 following LPS stimulation [37]. BHB‐treatment‐induced downregulation of the NLRP3 inflammasome pathway and shifted microglial polarization toward the neuroprotective M2 phenotype were also reported in the hippocampus of the HFFD/LPS‐induced sporadic AD model [38]. In addition, IF/BHB intervention has been shown to promote AB clearance from the brain [39], enhance BDNF expression in the hippocampus and improve cognitive function [40]. Our results extend these findings by directly linking the IF‐induced endogenous BHB to a lowered hippocampal amyloid burden, reduced oxidative stress, and decreased neuroinflammation, which led to increased BDNF expression and improved cognitive function.

Importantly, the greater benefits observed in the ADIFAE group suggest both synergistic and additive effects of IF and exercise. Several mechanisms plausibly underlie these findings. First, exercising in a fasted state accelerates the metabolic shift to ketosis by depleting hepatic glycogen more rapidly and increasing reliance on fatty acid oxidation and ketogenesis [41]. This is reflected in our data by markedly higher hippocampal BHB levels in ADIFAE versus ADIF or ADAE. Elevated BHB can directly inhibit NLRP3 inflammasome activation, which would account for the stronger suppression of caspase‐1 and pro‐inflammatory cytokines observed in the combined group. Second, although acute exercise is a mild physiological stressor that transiently increases ROS, the metabolic context determines the net effect. In the fed state, exercise‐induced ROS may transiently increase oxidative burden; by contrast, in a fasted (ketotic) state, the organism is prepared toward adaptive stress responses. IF and BHB promote antioxidant defenses and stress‐resilience, partly through epigenetic and signaling mechanisms, thereby lowering baseline oxidative stress [42, 43]. Exercising in the fasted state, therefore, likely elicits stronger endogenous antioxidant responses that neutralize ROS and reduce a key trigger for NLRP3 activation [44]. Consistent with this hypothesis, the ADIFAE group exhibited the largest reduction in MDA and the greatest increase in SOD activity, indicating improved redox homeostasis that could impede NF‐κB priming and inflammasome assembly.

However, the differential pattern—synergistic reduction in MDA but additive (nonsynergistic) effect on SOD activity—highlights the complexity of the oxidative stress adaptation to combined interventions. It likely suggests that fasted‐state exercise effectively targets multiple points in the oxidative stress pathway. While both interventions enhance the initial defense (SOD), the synergistic reduction in lipid peroxidation indicates a more profound impact on downstream processes or a more efficient prevention of the radical chain reaction that leads to lipid damage. This could involve enhanced activation of alternative antioxidant pathways (e.g., phase II enzymes like Nrf2‐mediated responses) or improved cellular clearance mechanisms for damaged lipids, beyond simply increasing the activity of the SOD enzyme itself. The additive effect pattern for SOD activity is consistent with the idea that both interventions likely target the primary enzymatic defense against superoxide radicals, potentially reaching a plateau where further enhancement is limited (ceiling effect). Future research could further investigate these potential mechanisms to elucidate the precise interplay between different antioxidant systems modulated by fasted‐state exercise.

The restoration of hippocampal BDNF levels in the combined intervention group is a likely consequence of these anti‐inflammatory and antioxidant effects. Reduced IL‐1β removes a well‐characterized inhibitory signal for BDNF transcription [4]. At the same time, both aerobic exercise and BHB directly stimulate BDNF expression by complementary mechanisms; exercise activates calcium‐dependent transcriptional cascades including CREB, which stimulates BDNF transcription [45], whereas BHB promotes a more permissive chromatin state via inhibition of histone deacetylases (HDACs), thereby enhancing BDNF gene expression [46]. Furthermore, aerobic exercise can also elevate BHB levels, which then indirectly contributes to BDNF induction [46]. The convergence of these pro‐BDNF signals in an environment with attenuated IL‐1β and oxidative stress provides a logical explanation for the enhanced recovery of BDNF and the parallel improvement in hippocampal‐dependent cognitive function observed in the ADIFAE group.

Our findings are consistent with a growing body of literature highlighting the benefits of combined metabolic interventions. For example, Miller et al. [47] observed that a 12‐week program combining exercise with a ketogenic diet improved insulin sensitivity and skeletal muscle mitochondrial function and efficiency, alongside weight loss, compared to exercise with a habitual mixed diet in healthy adults. The study by Pratchayasakul et al. [48] indicated that obese ovariectomized rats experienced improved metabolic and neurocognitive conditions when both caloric restriction and exercise were administered together, an effect greater than that of individual treatments. However, our results are contrary to the study by Albrahim et al. [22], who reported that combining exercise and IF did not provide additional benefits compared to either intervention alone on oxidative stress, neuroinflammation, and BDNF levels in the cortex of ovariectomized rats. This discrepancy likely relies on exercise duration and intensity (2–4 min × 15 min at 10–15 m/min vs. 1 min × 15 min at 18–25 m/min) as well as IF protocol (14 h fasting during the inactive day phase with water access ad libitum vs. 13 h fasting during the active dark phase without water access). Additional factors such as sex/hormonal status, brain region analyzed (hippocampus vs. cortex), and animal age (20‐month‐old vs. 8‐week‐old) could also account for the discrepancy.

Several limitations should be considered when interpreting our findings. First, we induced AD‐like pathology via bilateral intrahippocampal injection of Aβ_1–42_ to model amyloid‐driven neurotoxicity. While this approach isolates the effects of Aβ, it does not represent the full, progressive pathology of human AD (e.g., chronic plaque accumulation, tauopathy, and systemic changes). Second, the interventions were relatively short‐term (4 weeks), so the durability and long‐term efficacy of IF, aerobic exercise, or their combination remain unknown. Third, although the primary focus was on the effects of fasting and exercise interventions in fed and fasted states on amyloid burden, oxidative stress, and neuroinflammation in the hippocampus, the absence of systemic metabolic data limits the complete mechanistic interpretation. Finally, only male Wistar rats were used, which limits the generalizability of the results across sexes.

In summary, our study demonstrates that the timing of exercise relative to feeding can influence metabolic and signaling outcomes. Although IF × exercise interaction effects were not uniform across all measures, the overall pattern indicates that performing aerobic exercise in a fasted state provides complementary metabolic, anti‐inflammatory, and cognitive benefits that exceed those of either intervention alone. These findings suggest that combining IF with regular aerobic exercise may be an effective multimodal strategy for mitigating Aβ‐related hippocampal dysfunction.

## Author Contributions

Investigation, conceptualization, methodology: Davar Khodadadi, Taner Atasoy, and Heidar Sajedi. Data curation: Taner Atasoy, Davar Khodadadi, and Mohammad Babaei. Formal analysis: Burak Tozoğlu, Davar Khodadadi, Mehmet Şirin Güler, and Serkan Tevabil AKA. Writing – original draft: Taner Atasoy, Heidar Sajedi, Burak Tozoğlu, Mehmet Şirin Güler, and Serkan Tevabil AKA. Writing – review and editing: Davar Khodadadi, Heidar Sajedi, and Mohammad Babaei. Project administration: Davar Khodadadi.

## Funding

The authors received no specific funding for this study.

## Disclosure

All scientific content was created and reviewed by the authors. All authors reviewed and approved the final manuscript.

## Conflicts of Interest

The authors declare no conflicts of interest.

## Supporting Information

Additional supporting information can be found online in the Supporting Information section.

## Supporting information


**Supporting Information** Figure 1. Figure 5A raw blots. Figure 2. Additional representative western blot experiments in the rest of animals.

## Data Availability

The data that support the findings of this study are available from the corresponding author upon reasonable request.
