# Supplementary figures and images for: Intermittent Fasting Potentiates Aerobic Exercise to Reduce Hippocampal Amyloid Burden and Oxidative Stress via Suppression of NF‐κB/NLRP3 Signaling in an Aβ‐Injected Rat Model
Source: Oxid Med Cell Longev. 2026 May 18;2026:9921337. doi: 10.1155/omcl/9921337 (PMC13184385; doi:10.1155/omcl/9921337)

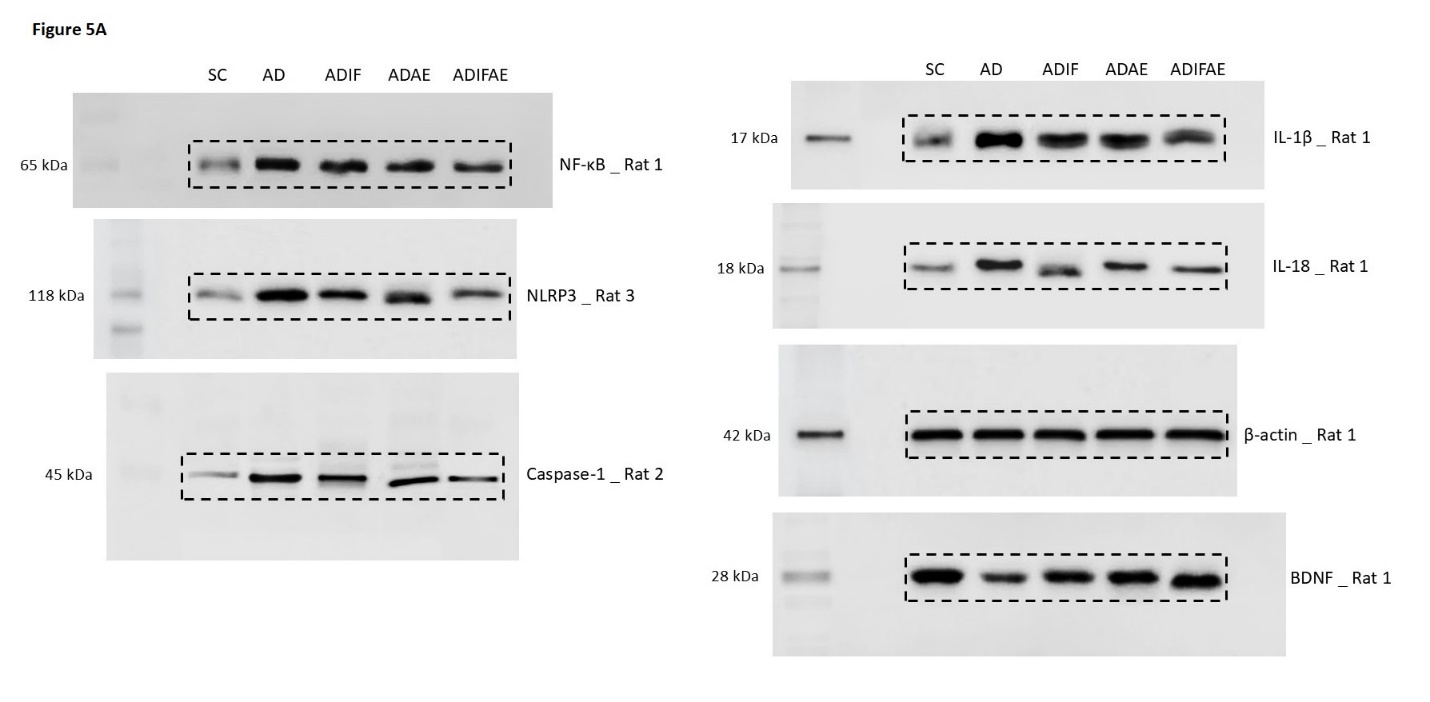


**Supplementary Figure 1.**


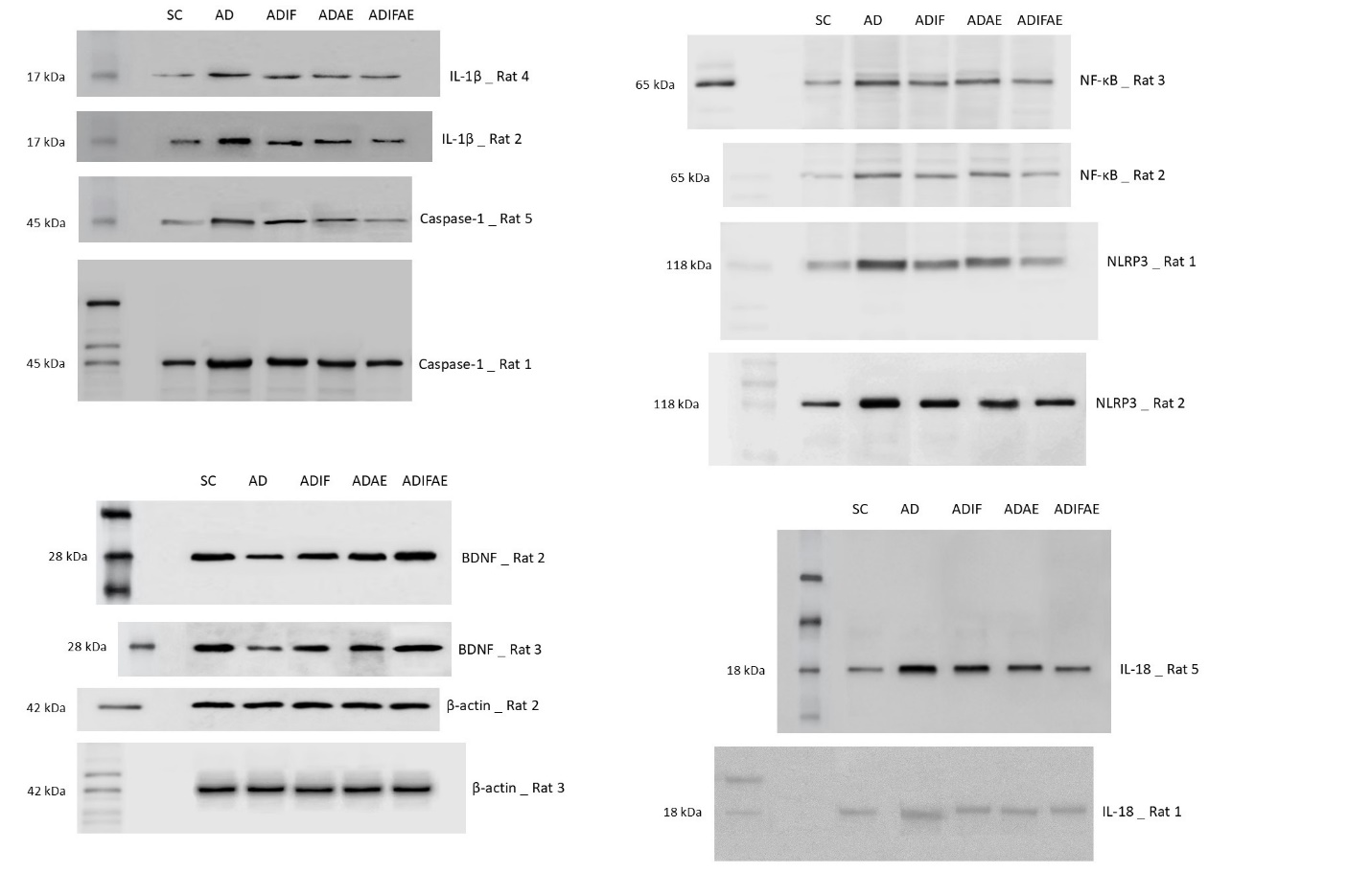


**Supplementary Figure 2.**

Supplement: Supplementary file 1 — Supporting Information Figure 1. Figure 5A raw blots. Figure 2. Additional representative western blot experiments in the rest of animals. [file OMCL-2026-9921337-s001.docx]
